# Variations in age‐ and sex‐specific survival rates help explain population trend in a discrete marine mammal population

**DOI:** 10.1002/ece3.4772

**Published:** 2018-12-19

**Authors:** Mònica Arso Civil, Barbara Cheney, Nicola J. Quick, Valentina Islas‐Villanueva, Jeff A. Graves, Vincent M. Janik, Paul M. Thompson, Philip S. Hammond

**Affiliations:** ^1^ Sea Mammal Research Unit, Scottish Oceans Institute University of St Andrews St Andrews UK; ^2^ Lighthouse Field Station, Institute of Biological and Environmental Sciences University of Aberdeen Cromarty UK; ^3^ Duke University Marine Laboratory Nicholas School of the Environment Beaufort North Carolina; ^4^ CONACYT, Universidad del Mar, Instituto de Genética Ciudad Universitaria Oaxaca México; ^5^ Centre for Biological Diversity University of St Andrews St Andrews UK

**Keywords:** calf survival, capture–recapture, mortality rate, population dynamics, sex‐specific survival, *Tursiops*

## Abstract

Understanding the drivers underlying fluctuations in the size of animal populations is central to ecology, conservation biology, and wildlife management. Reliable estimates of survival probabilities are key to population viability assessments, and patterns of variation in survival can help inferring the causal factors behind detected changes in population size. We investigated whether variation in age‐ and sex‐specific survival probabilities could help explain the increasing trend in population size detected in a small, discrete population of bottlenose dolphins *Tursiops truncatus* off the east coast of Scotland. To estimate annual survival probabilities, we applied capture–recapture models to photoidentification data collected from 1989 to 2015. We used robust design models accounting for temporary emigration to estimate juvenile and adult survival, multistate models to estimate sex‐specific survival, and age models to estimate calf survival. We found strong support for an increase in juvenile/adult annual survival from 93.1% to 96.0% over the study period, most likely caused by a change in juvenile survival. Examination of sex‐specific variation showed weaker support for this trend being a result of increasing female survival, which was overall higher than for males and animals of unknown sex. Calf survival was lower in the first than second year; a bias in estimating third‐year survival will likely exist in similar studies. There was some support first‐born calf survival being lower than for calves born subsequently. Coastal marine mammal populations are subject to the impacts of environmental change, increasing anthropogenic disturbance and the effects of management measures. Survival estimates are essential to improve our understanding of population dynamics and help predict how future pressures may impact populations, but obtaining robust information on the life history of long‐lived species is challenging. Our study illustrates how knowledge of survival can be increased by applying a robust analytical framework to photoidentification data.

## 1 INTRODUCTION

Knowing whether populations are increasing or declining and understanding the drivers behind such fluctuations are important issues in ecology, conservation biology and wildlife management (Eberhardt, 1985; Galimberti, Sanvito, Boitani, & Fabiani, 2001; Williams, Nichols, & Conroy, 2002). Patterns in the variation of survival rates are important indicators in population dynamics (e.g., Kraus et al., 2005; Altwegg, Crawford, Underhill, & Williams, 2008), and reliable estimates of survival probabilities are required to assess and determine the viability of populations (Doak, Kareiva, & Klepetka, 1994; White, Franklin, & Shenk, 2002). Mammalian age‐specific survival is typified by a U‐shaped mortality curve, characterized by high rates in young animals and low rates in adults that increase toward the maximum age (Caughley, 1966). Males tend to have higher mortality rates than females (Trivers, 1972). Variations in age‐ and sex‐specific survival rates modulate population dynamics; adult survival typically has a greater effect on population trends than juvenile survival (Eberhardt, 2002; Fowler, 1987).

In a conservation context, monitoring to determine trends in population size is crucial to inform assessments of wildlife populations (e.g., IUCN, 2017). The cause of a trend may be understood from knowledge of human‐induced mortality, for example, but in other cases the cause may not be so easily revealed. In such cases, estimates of fecundity and survival can play an important role in helping to understand which element(s) of life history may be responsible for changes in population size (e.g., Gaillard, Festa‐Bianchet, & Yoccoz, 1998; Currey et al., 2011).

Long‐term individual‐based studies are an effective tool to estimate population parameters from discrete populations in which individuals can be repeatedly captured over time (Clutton‐Brock and Sheldon, 2010), and are particularly important in long‐lived species for which the ability to detect changes in demographic parameters may require decades of data. Capture–recapture analyses of individual‐based data have been used extensively in ecology (Burnham, Anderson, White, Brownie, & Pollock, 1987; Cormack, 1964) to estimate survival probabilities (e.g., Gaillard et al., 1998; Altwegg et al., 2008). Obtaining robust estimates of survival probabilities in cetacean populations remains challenging, and estimates of age‐ and sex‐specific survival are scarce (e.g., Bradford et al., 2006; Currey et al., 2009; Ramp, Bérubé, Palsboll, Hagen, & Sears, 2010).

**Figure 1:**
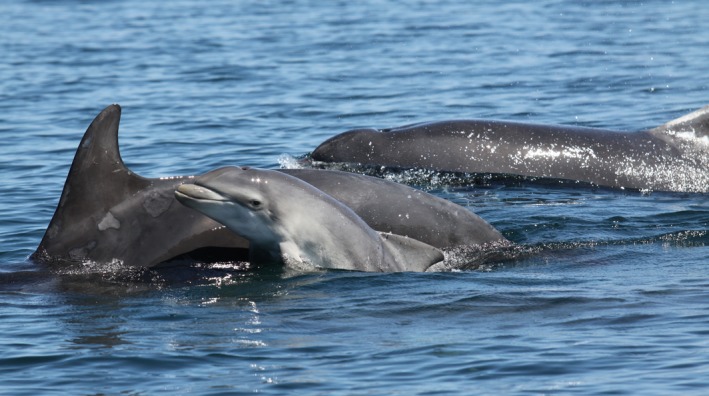
Bottlenose dolphins from the east coast of Scotland (photograph by Mònica Arso Civil, taken during this study)

Our focus here is the east coast of Scotland bottlenose dolphin (*Tursiops truncatus*) population (Figure 1), which long‐term photoidentification monitoring since 1989 indicates is increasing, especially since around 2000 (Cheney, Graham, Barton, Hammond, & Thompson, 2018; Wilson, Hammond, & Thompson, 1999; Wilson, Reid, Grellier, Thompson, & Hammond, 2004). Survival rates have been estimated for this population using eight (Sanders‐Reed, Hammond, Grellier, & Thompson, 1999) and 13 (Corkrey et al., 2008) years of data collected in the 1990s to early 2000s. Data for both studies indicated a greater probability of a population decline than of an increase. However, this population expanded its distributional range during the 1990s (Wilson et al., 2004), meaning that these estimated declines are likely to be confounded with temporary emigration (Corkrey et al., 2008).

Here, we use a 27 year‐long dataset of individual capture histories to investigate variations in age‐ and sex‐specific survival rates in this small, discrete population of bottlenose dolphins. We explore whether changes in juvenile/adult survival could help explain the observed increase in population size and investigate variation in calf survival. We first estimate annual survival of juvenile/adult dolphins using robust design capture–recapture models, which incorporate the estimation of temporary emigration, including assessing the support for a trend over time. We then explore whether there is evidence for sex‐specific survival using multistate capture–recapture models. We estimate calf survival during the first 3 years of life for a subset of dolphins followed since their year of birth using age‐specific models, and investigate whether survivorship of first‐born calves was different from calves born subsequently.

## 2 MATERIALS AND METHODS

### 2.1 Photoidentification data

**Figure 2:**
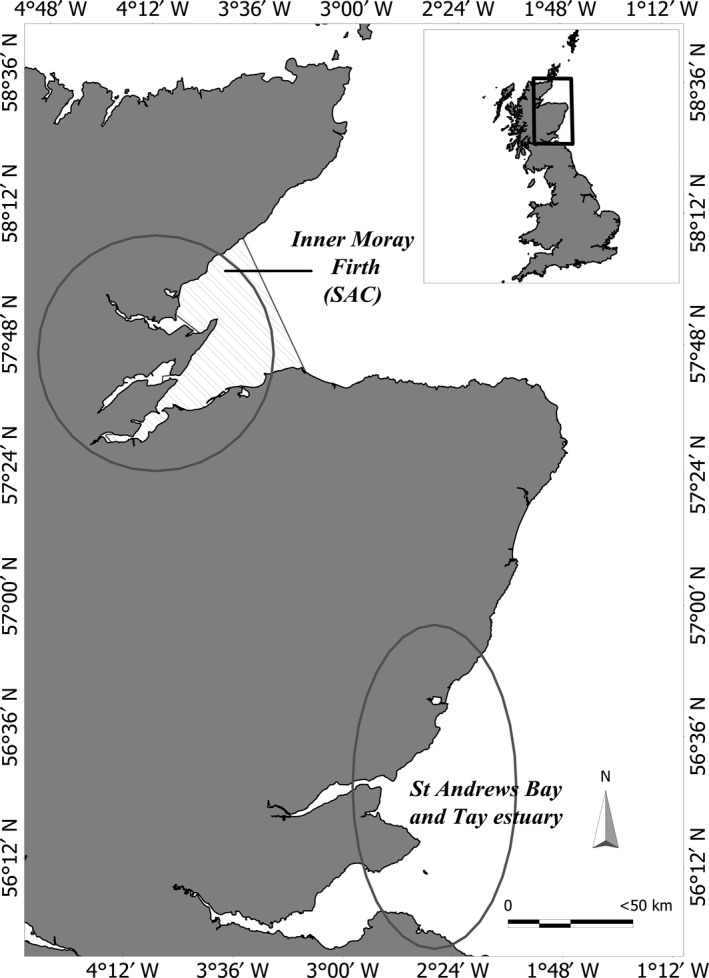
Map showing the two main study areas (circles) located in the Moray Firth Special Area of Conservation (SAC; dashed area) and in St Andrews Bay and the Tay estuary

Boat‐based surveys were conducted off the east coast of Scotland (Figure 2) between May and September every year from 1989 to 2015 to collect photoidentification data of bottlenose dolphins following standardized protocols (Cheney et al., 2014; Islas‐Villanueva, 2009; Quick & Janik, 2008). Sampling effort occurred in two main areas at opposite ends of the population's main distributional range; in the Moray Firth Special Area of Conservation (SAC) effort was consistent over the study period (Cheney et al., 2014), while in St Andrews Bay and the Tay estuary effort was variable from 1997 to 2007 and consistent from 2009 onwards (Cheney et al., 2013; Islas‐Villanueva, 2009; Quick & Janik, 2008). Individual dolphins were identified from high‐quality photographs (Wilson et al., 1999) based on unique markings in the dorsal fin (Würsig & Jefferson, 1990), and matched against a catalogue of previously identified bottlenose dolphins from the east coast of Scotland. Because bottlenose dolphin calves do not tend to have permanent marks that can be tracked across years, individuals in the first 3 years of life were aged and identified based on body size, skin coloration, presence of fetal folds and repeated association with a known adult dolphin (i.e., the mother; Grellier, Hammond, Wilson, Sanders‐Reed, & Thompson, 2003; Arso Civil, Cheney, Quick, Thompson, & Hammond, 2017). Capture histories of marked juveniles (at least 4 years of age) and adults (i.e., dolphins with distinctive long‐lasting nicks on the trailing edge of the dorsal fin) and of calves were constructed to model survival probabilities. The sex of individual dolphins was determined from photographs of the genital area, molecular analysis of biopsy samples (Islas‐Villanueva, 2009) or the identification of mother–calf pairs.

### 2.2 Modeling age‐ and sex‐specific survival

The open population Cormack‐Jolly‐Seber (CJS) model is typically used to estimate survival probabilities from capture–recapture data (e.g., Pollock, Nichols, Brownie, & Hines, 1990). Capture–recapture models rely on a number of assumptions about the captured individuals and about the probability of capture, which can generate bias in the estimated model parameters if violated (Hammond, 2010; Pollock et al., 1990). Conventional open population models assume, among other things, that any emigration from the study population is permanent (Kendall, Nichols, & Hines, 1997), an assumption that is violated when animals transit through an area and are not available for capture after first release (Pradel, Hines, Lebreton, & Nichols, 1997) or when animals range beyond the study area and are unavailable for capture on some occasions (i.e., temporary emigration; Kendall et al., 1997).

Our study population expanded its distributional range in the 1990s (Wilson et al., 2004), but sampling effort only gradually expanded outside the initial study area from the end of the 1990s. Combined with variability in movement patterns among individuals in the population (Cheney et al., 2013), this means that temporary emigration is likely to be a feature of our study, as suggested by Corkrey et al. (2008). Failure to account for this may lead to bias. Methods to determine the sex of individuals can also be a source of bias; in cetaceans assigning sex reliably typically requires repeatedly resighting individuals, positively biasing the survival of sexed individuals simply because they have been sighted for longer than unsexed animals (Nichols, Kendall, Hines, & Spendelow, 2004).

Prior to survival modeling, the key assumptions about the probability of capture (Lebreton, Burnham, Clobert, & Anderson, 1992) were explored in each dataset using goodness‐of‐fit tests in program U‐CARE (Choquet, Lebreton, Gimenez, Reboulet, & Pradel, 2009). Assumptions were satisfied for the calf and the multistate (sex‐specific) juvenile/adult datasets, but showed evidence for trap‐dependence and transience in the single‐state juvenile/adult dataset (see below for dataset details), stressing the need to use a modeling approach which could incorporate temporary emigration and heterogeneity in the capture probabilities. A small level of overdispersion was detected in the single‐state juvenile/adult dataset, and model statistics were adjusted by the estimated overdispersion factor (ĉ = 1.629; Choquet et al., 2009). Model selection was based on the Akaike Information Criterion (AIC; Akaike, 1973) adjusted for small samples (AICc) for the calf and the multistate juvenile/adult datasets, and on the Quasi Akaike Information Criterion (QAIC_c_) for the single‐state juvenile/adult dataset (Burnham & Anderson, 2002). Model structures and parameters were specified and run using the package RMark (Laake, 2013) in R (R Core Team, 2016), and program MARK (White & Burnham, 1999).

#### 2.2.1 Juvenile/Adult survival: Robust design models

Robust design (RD) models (Kendall & Nichols, 1995; Kendall et al., 1997; Pollock, 1982) were used to estimate survival probabilities of juvenile/adult dolphins (i.e., non‐calf), combining open and closed population models with estimators that incorporate temporary emigration. Each annual field season represented a primary sampling occasion and months within each season were treated as secondary sampling occasions. Parameters estimated by RD models include the probabilities of survival, temporary emigration, capture and recapture. Survival was first set to be constant in all models to obtain an average survival estimate for juveniles/adults.

The probability of temporarily emigrating was modeled as the probability that an animal was unavailable for capture during a primary period (i.e., a given year), given that it was available (γ’’) or unavailable (γ’) in the previous primary period. Random temporary emigration is characterized by the probability of emigrating not depending on whether or not an animal was previously available (γ’’ = γ’). Markovian temporary emigration occurs when the probability of emigrating depends on whether an animal was previously available or not (γ’’ ≠ γ’). Models with random or Markovian temporary emigration were fitted, with probabilities allowed to be constant or time‐dependent, in which case constraints were applied to allow identifiability of parameters (Kendall et al., 1997). No‐movement models (γ’’ = γ’ = 0) were included for comparison.

Recapture probability was assumed to equal capture probability for all models because photoidentification does not involve handling of the animals and is not expected to cause any behavioral changes in the captured individuals. Capture probabilities were allowed to vary over both primary and secondary periods in all models because preliminary analysis showed less support for models restricting capture probabilities to vary between years but not within year, or not to vary at all. Models incorporating individual heterogeneity were also considered (π; Pledger, 2000), in which the population was assumed to comprise a mixture of two types of individual with different probability of capture, with probability π of being in the first type. Using the most supported model regarding capture and temporary emigration probabilities, a trend in survival probability over the study period was then explored.

#### 2.2.2 Sex‐specific survival: Multistate models

To account for uncertainty in sex assignment and thus reduce bias in estimates of survival (Nichols et al., 2004; Pradel et al., 2008), multistate (MS) models (e.g., Hestbeck, Nichols, & Malecki, 1991; Brownie, Hines, Nichols, Pollock, & Hestbeck, 1993) were used to estimate the sex‐specific probability of survival of juvenile/adult dolphins. Sightings of individuals made during the same year were pooled together in one sampling occasion. States were defined as male (M), female (F), or unknown (U) if sex had not been determined. When first sighted, an individual was recorded as unknown (U) until the sex was determined (M or F), and then remained in that state for all following re‐sightings. Parameters estimated in the MS models included the probabilities of recapture and survival by state (i.e., by sex) and transition probabilities between states. Individuals could only transition from state U to either state M or F, fixing all other transition probabilities at zero. Transition probabilities in years when no males or females were sexed were fixed at zero. A set of candidate models was created to investigate the effects of no variation, time‐dependence, time trend, and state (sex‐specific*:* *M *≠ *F *≠ U, or the combinations *M* = U ≠ F, *F* = U ≠ M) on survival, recapture and transition probabilities. Models with constant transition probabilities over time were not considered because the number of animals sexed varied markedly among years.

#### 2.2.3 Calf survival: Age‐specific CJS models

Age‐specific CJS models were used to estimate the probability of survival of dolphins during the first 3 years of life. Because the probability of sighting a calf was highly dependent on sighting the mother (Grellier et al., 2003), we only included calves with a known mother and year of birth, and whose mothers were seen in the year of birth and in the three subsequent years. Recapture probabilities were allowed to be constant or to vary among years. Survival was modeled as constant for all ages, separately for 1st, 2nd and 3rd year, or for 1st year and 2nd/3rd year as a single parameter. Models were also fitted to investigate whether survivorship of first‐born calves was different from calves born subsequently.

## 3 RESULTS

### 3.1 Juvenile/Adult survival

**Figure 3:**
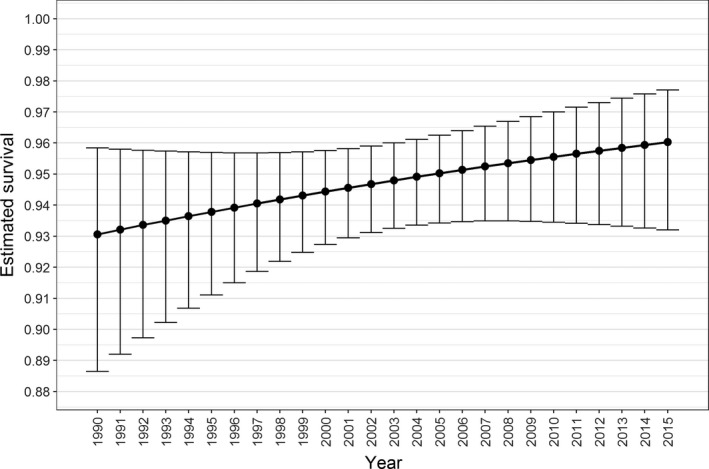
Time trend in juvenile/adult estimated survival probability (with associated 95% confidence intervals) from the most supported RD model

In total, 205 juvenile/adult marked bottlenose dolphins were identified between 1989 and 2015. Model selection favored models incorporating Pledger (2000) heterogeneity mixture parameters over those without it. Including a trend in the probability of survival (model 1 in Supporting Information Table S1) greatly improved the model fit (ΔQAICc = 9.6, compared to the equivalent model with time‐invariant survival, model 2). This model had Markovian temporary emigration with a very low constant probability of emigrating (γ’’) of 0.017 (95% CI: 0.006–0.047) and a high constant probability of remaining an emigrant (γ^’^) of 0.712 (95% CI: 0.358–0.915). The estimated survival probability increased from 0.931 (95% CI 0.886–0.958) in 1990 to 0.960 (95% CI 0.932–0.977) in 2015 (Figure 3). The next best‐supported model incorporated a constant survival probability of 0.948 (95% CI 0.933–0.959; model 2 in Supporting Information Table S1) and very similar estimates of Markovian emigration to the model with a time trend in survival.

To investigate if the detected trend in survival was more likely to be a result of a change in adult or juvenile survival, the top two most supported models in Supporting Information Table S1 were fitted to a restricted dataset of adults only. This dataset excluded the first 6 years of sightings for those dolphins classified as juveniles when first seen, and the first 3 years of sightings for those classified as subadults (based on body size). For this adult‐only dataset, the model with constant survival probability received more support (ΔQAICc = 4.4) compared to the one that included a trend in survival.

### 3.2 Sex‐specific survival

**Figure 4:**
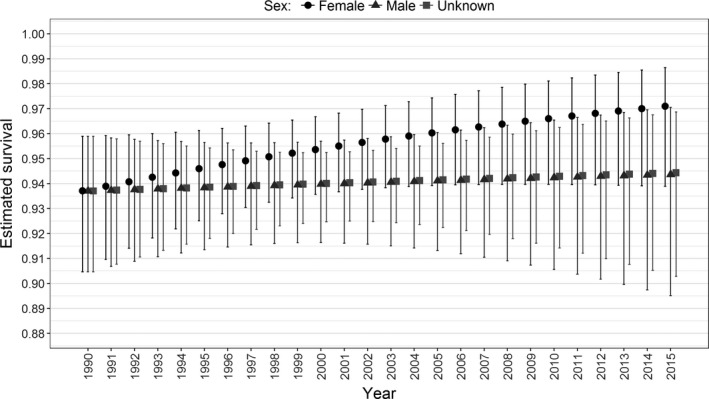
Time trend in sex‐specific estimated survival probabilities with associated 95% confidence intervals for juvenile/adult bottlenose dolphins. The estimated parameters are derived from multistate model number 5 in Supporting Information Table S2

The marked juvenile/adult dolphins identified between 1989 and 2015 included 43 males, 66 females and 96 animals of unknown sex. Multistate (MS) models that allowed transition and capture probabilities to vary among years but not between sexes had better support from the data than other models (Supporting Information Table S2). In the top eight MS models, all within a ΔAICc < 4 indicating some support from the data, survival was parameterized in a variety of ways including constant, sex‐specific, and with and without a temporal trend. Within this suite of models, there was most support for survival being different between females and males/unknown sex but the model with constant survival also received considerable support (Supporting Information Table S2). Models incorporating a sex‐specific temporal trend in survival had relatively weak support (Supporting Information Table S2) but showed a more pronounced trend in survival for females than for males and animals of unknown sex (Figure 4). Estimated survival was higher for females (0.962; 95% CI 0.941–0.976) than males (0.942; 95% CI 0.904–0.966; Table 1).

**Table 1:** Estimates of time‐invariant survival probability with associated 95% Confidence Interval (CI) from the best‐supported models of age‐specific survival, sex‐specific survival and the survival of first‐ or subsequently born calves (model details given in Supporting Information Table S1–S3)

Age class	Group	Modeling approach	Survival	95% CI
Juvenile/adult	All	Robust design	0.948	0.933–0.959
	Female	Multistate	0.962	0.941–0.976
	Male		0.942	0.904–0.966
	Unknown sex		0.939	0.923–0.952
Calf—1st year	All	Age‐CJS	0.865	0.785–0.919
Calf—2nd year			0.981	0.797–0.998
Calf—3rd year			0.883	0.708–0.959
Calf—1st year	First‐born		0.836	0.697–0.918
	Subsequently born		0.880	0.789–0.935
Calf—2nd year	First‐born		0.976	0.744–0.998
	Subsequently born		0.983	0.814–0.999
Calf—3rd year	First‐born		0.853	0.619–0.954
	Subsequently born		0.894	0.725–0.964

### 3.3 Calf survival

In total, 116 calves with known year of birth and known mother were identified between 1990 and 2015. Of these, 56 calves had mothers sighted in the 3 years following birth. Models restricting the recapture probability to be invariant received a lot more support than those allowing it to vary among years and ages, resulting in a very high recapture probability of 0.954 (95% CI: 0.914–0.975, model 1 in Supporting Information Table S3). Models in which survival probability was constant, different for each age class or different between age 1 and ages 2/3 combined were all well supported by the data (models 1–3). Survival probabilities of calves were lowest in the 1st year, increased in the 2nd year and decreased in the 3rd year (Table 1). Models in which the survival of first‐born calves was different from calves born subsequently received considerable support from the data but not as much as models that did not incorporate this (models 4 and 5 in Supporting Information Table S3). Estimated survival of first‐born calves was lower than that of later‐born calves at any age (Table 1).

## 4 DISCUSSION

In this analysis of age‐ and sex‐specific survival in a small, discrete coastal bottlenose dolphin population, we present evidence for variation in juvenile/adult survivorship over two and a half decades that is consistent with an increasing population. We also provide the first estimates of calf survival probabilities for this population, adding to the very few reliable estimates available for this or any other cetacean species.

### 4.1 Increasing juvenile/adult survival

Our estimate of time‐invariant survival probability for juveniles/adults in the Scottish east coast bottlenose dolphin population (0.948, 95% CI: 0.933–0.960) is comparable to estimates reported for these age classes in other coastal bottlenose dolphin populations (Currey et al., 2011; Smith, Pollock, Waples, Bradley, & Bejder, 2013; Speakman, Lane, Schwacke, Fair, & Zolman, 2010). Our estimate is higher than those previously reported for our population by Sanders‐Reed et al. (1999; 0.942, *SE* = 0.015) and Corkrey et al. (2008) (0.93, *SE *= 0.029), using data from 1990–1997 and 1990–2002, respectively; both of these studies suggested that a population decline was more probable than an increase. However, any prediction of decline was likely confounded with temporary emigration (Corkrey et al., 2008) because of the population's range expansion (Wilson et al., 2004). Our analysis is based on a much larger dataset (27 years compared to 13 years) that encompasses changes in the population's range and the subsequent changes in sampling effort. By explicitly modeling temporary emigration, we minimized bias in the estimates of survival probability that could result by ignoring this.

It is generally accepted that in slow‐growing populations of long‐lived species, adult survival has more influence on population growth than reproduction (Crone, 2001; Oli & Dobson, 2003). In a number of cetacean populations, trends in probabilities of survival have been linked to changes in population size (e.g., Kraus et al., 2005; Ford, Ellis, Olesiuk, & Balcomb, 2010; Currey et al., 2011; Gormley et al., 2012). Our results showed very strong support for an increase in juvenile/adult survival over two and a half decades, most likely caused by a change in juvenile survival, which translates into a 45% decrease in mortality rate (from 6.9% in 1990 to 4.0% in 2015). This change is likely to be responsible, at least partially, for the increase in population size identified by Cheney et al. (2014).

However, some studies highlight the importance of reproduction versus adult survival in shaping population dynamics (Manlik et al., 2016; Mitchell, Pacifici, Grand, & Powell, 2009). Arso Civil et al. (2017) estimated a fecundity rate for the study population similar to those reported for other stable bottlenose dolphin populations but Cheney (2017) found an increasing trend in reproductive rates using a restricted dataset of reproductive females utilizing the Moray Firth SAC between 2001 to 2016. Therefore, a parallel change in fecundity rate cannot be ruled out.

What could have caused the observed increase in survival probability? A decline in the use of illegal gill nets in the inner Moray Firth, which was an identified cause of mortality for this population during the 1990s (Butler et al., 2017), could have contributed to the increased survival, as identified for Hector's dolphins (*Cephalorhynchus hectori*) in New Zealand (Gormley et al., 2012). Wilson et al. (2004) hypothesized that the population's range expansion in the 1990s was more likely to be explained by changes in prey resources than an increase in population size, partially because of the apparent negative population growth at the time. This is supported by more recent studies that have identified high use areas, including foraging areas, outside the inner Moray Firth, which was the main area of distribution before the range expansion (Arso Civil, 2014; Pirotta et al., 2013). Also, dolphin movements across the distributional range are variable, with lower rates of exchange between the most geographically distant areas (Cheney et al., 2013). If, as seems likely, the range expansion has resulted in more prey resources being available to the population, this could have led to improved fitness of individual dolphins, and, consequently, an improved probability of survival in the population.

Demographic parameters such as survival and reproductive rates may change in other populations of mobile species as a consequence of environmental change, disturbance or management action, which may result in changes in the availability of or access to prey resources and/or a reduction in anthropogenic mortality sources. A reduction in foraging activity due to human pressure (e.g., Pirotta, Merchant, Thompson, Barton, & Lusseau, 2015, Williams, Lusseau, & Hammond, 2006) or a displacement of individuals from foraging areas (Pirotta et al., 2013) that affects their energy intake could have long‐term population consequences through changes in individual vital rates (New et al., 2013). Changes in prey availability caused by environmental variability have been linked to changes in the survival and reproduction rates of killer whales (*Orcinus orca*) (e.g., Ford et al., 2010, Ward, Holmes, & Balcomb, 2009), while anthropogenic modifications to habitat have been linked to in increased age‐specific mortality rates in another population of bottlenose dolphins (e.g., Currey et al., 2011). A decrease in mortality pressure due to management actions can result in significant changes in survival (e.g., Gormley et al., 2012) and increases in population size, as in the case of some whale populations recovering from past overexploitation as they reoccupy territories and exploit existing resources (Branch, Matsuoka, & Miyashita, 2004; Pallin et al., 2018).

### 4.2 Sex‐specific survival

Available estimates of sex‐specific survival/mortality for bottlenose dolphins are scarce but all suggest higher mortality rates for males (e.g., Scott, Wells, & Irvine, 1990; Stolen & Barlow, 2003; Currey et al., 2009). Our estimate of male mortality rate (5.8% per annum) was almost double that for females (3.8% p.a.; Table 1) but, although sex‐specific survival models were most supported by the data, support for them was not particularly strong. Mortality rate, calculated here as 1 − *apparent* survival rate, is a combination of the true mortality and any permanent emigration confounded with mortality. However, there is no evidence of permanent emigration in this population. Comparison of photoidentification data between the east and west coasts of Scotland showed no matches, suggesting no permanent or temporary movement of animals between those two areas (Cheney et al., 2013). Long‐distance movements reported by Robinson et al. (2012) between the east and west coast of Scotland and Ireland were of an isolated group of individuals never seen in the east coast previously or subsequently to their encounter and were thus never part of the east coast of Scotland catalogue. These results are in accordance with those from genetic analysis which show some but not complete isolation between animals in this population and those found elsewhere in Britain (Parsons, Noble, Reid, & Thompson, 2002; Thompson et al., 2011).

Sex‐differentiated mortality rates in mammals have been mainly attributed to sexual selection costs (Promislow, 1992; Trivers, 1972). In cetacean species, these include costs associated with male selection in polygynous mating systems (Ralls, Brownell, & Ballou, 1980), sex differences in ranging patterns (Stolen & Barlow, 2003), and sex differences in the accumulation of toxic burdens with age (Schwacke et al., 2002). Male bottlenose dolphins in the study population show a significantly higher percentage of body scarring and dorsal fin nicks compared to females (Marley, Cheney, & Thompson, 2013), which has been related to male–male competition (Scott, Mann, Watson‐capps, Sargeant, & Connor, 2005). If males move spatially and temporally more than females, as observed in other bottlenose dolphin populations (Möller & Beheregaray, 2004; Scott et al., 1990), it could compromise their fitness and hence survival, assuming an increase in energetic costs. Jepson et al. (2016) report higher levels of polychlorinated biphenyls (PCBs) from stranded and biopsied male bottlenose dolphins from the NE Atlantic, compared to females, which transfer part of their toxic burden to their offspring through lactation, although the exact role of contaminants in the survival of marine mammals remains unclear (Ross, 2002).

Our results also showed some support for a female‐driven increase in juvenile/adult survival, which suggests that the hypothesized improved fitness in juveniles/adults following the range expansion was more pronounced in females compared to males and dolphins of unknown sex. The latter group is likely to include a higher proportion of juveniles and adult males than females because of sex assignment based on photographs. Estimating sexual selection costs is challenging but sex differences in ranging patterns could be investigated to improve understanding of why there may have been a sex‐differentiated trend in survival in this population.

### 4.3 Calf survival

First‐year survival estimates are available for a few well‐studied bottlenose dolphin populations (Currey et al., 2009; Mann, Connor, Barre, & Heithaus, 2000; Stolen & Barlow, 2003; Wells & Scott, 1990) and compare well with our estimate (0.865, 95% CI: 0.785–0.919). We found that second‐year survival increased to 0.981 but third‐year survival decreased to 0.708, which would not be expected if the age‐specific mortality curve that is characteristic of long‐lived mammals were strictly U‐shaped (Caughley, 1966). This drop in estimated survival in the third year could be a result of increased mortality when calves are weaned and become independent from their mothers, which starts to occur in their third year of life (Grellier et al., 2003; Mann et al., 2000). Alternatively, it could be a result of mark loss. Young bottlenose dolphins have typically not yet acquired permanent marks; a three‐year old calf associating less with its mother (Grellier et al., 2003) could be lost from the marked population because it was unable to be identified. Three‐year inter‐birth intervals occur less than 20% of the time in this population (Arso Civil et al., 2017) but the mothers of nine of the sixteen calves not captured in their third year of life were sighted that year accompanied by a new calf. The extent to which our estimate of third‐year survival is negatively biased by mark loss is unknown but obtaining estimates of age‐specific survival probabilities in older calves is likely to be problematic in other studies relying on photoidentification to recognize individuals.

Censoring the data to include only calves whose mothers were seen during the 3 years after calf birth could overestimate calf survival, as calves are very likely to die if the mother dies in their first year, and more likely to die if the mother dies in their second year and before weaning (Noren & Edwards, 2007). The maximum bias in first‐ or second‐year calf survival would be equivalent to the mortality rate of adult females (3.8%), which would result in a first‐year calf survival as low as 0.832.

Cheney, Wells, Barton, and Thompson (2017) have shown that first‐born calves in the study population are shorter in length than calves born subsequently and found weak evidence for fitness consequences on first‐year calf survival. Our results also suggest, although not strongly, that first‐born calves have lower survival rates than later‐born siblings, especially during the first year of life. This is consistent with the available information from a limited number of other well‐studied populations of bottlenose dolphins (Henderson, Dawson, Currey, Lusseau, & Schneider, 2014; Mann et al., 2000). Suggested reasons behind a differentiated mortality in first‐born calves include poor calf condition (Cheney et al., 2017; Mann & Watson‐Capps, 2005; Rowe, Currey, Dawson, & Johnson, 2010), individual differences among mothers in successfully rearing calves (Henderson et al., 2014) and the detrimental effect of organochlorine compounds transferred from the mother, which is higher in first‐born calves (Hall et al., 2006; Wells et al., 2005).

### 4.4 Applications

Our results inform assessment of the conservation status of this population, a requirement under the European Habitats Directive (Council Directive 92/43/EEC). As mentioned above, one cause of the increasing survival rates could have been the decline in the use of gillnets in the inner Moray Firth in the 1990s. Knowledge of how survival rates have changed in the past will inform our understanding and assessment of how future pressures, such as the development of offshore wind energy, may impact the east coast population of bottlenose dolphins. Population viability analysis (PVA; e.g., Lacy, 2000) is a useful framework for exploring such impacts. The availability of new information on survival presented here and on reproductive rates (Arso Civil et al., 2017) provides an excellent basis for revisiting the PVA that was attempted 20 years ago (Sanders‐Reed et al., 1999).

More generally, information on survival will become an increasingly important part of the assessment of conservation status and the impact of human activities on coastal cetaceans. Information of sufficient robustness and detail, and over a sufficient time period, has hitherto been sparse (e.g., Currey et al., 2009; Gormley et al., 2012). But as time series of photoidentification data increase in intensity and length in ongoing studies, results from survival analyses will become more available for input into the assessment process (such as through PVAs). Our study serves as a useful indication of the information that can be made available with sufficient data and a robust analytical framework. Coastal marine mammal populations are globally subject to the impacts of environmental change and increasing anthropogenic disturbance but also to the effects of management measures to reduce human impact. Obtaining robust estimates of age‐ and sex‐specific survival will help understand how populations are responding to these changes.

## 5 CONCLUSIONS

In generating robust estimates of survival for a small and discrete population of bottlenose dolphins, we showed very strong support for an increase over time in juvenile/adult survival, fairly strong support for age‐specific calf survival and sex‐specific juvenile/adult survival, and weaker support for females driving the trend in juvenile/adult survival and an impact of birth order on calf survival. These results inform our understanding of reasons for an observed change in population size and provide essential information to parameterize future population assessments (PVAs) to explore the impact of human activities. Our study serves as a useful indication of the information that can be made available with sufficient data and a robust analytical framework, to ultimately help understand how populations may respond to the impacts of environmental changes, anthropogenic pressure, as well as management measures implemented to reduce human impacts.

## CONFLICT OF INTEREST

None declared.

## AUTHOR CONTRIBUTIONS

MAC and PSH conceived the initial ideas and designed methodology; MAC, NQ, BC, VI, and VMJ collected the photo‐ID data; VMJ and VI collected biopsies for sexing; VI and JAG conducted the analysis of sex animals; MAC, BC, VI, and NQ processed all photo‐ID data; PMT contributed to the interpretation of data for the work and to the analysis of the data; MAC analyzed the data; MAC and PSH led the writing of the manuscript. All authors contributed critically to the drafts and gave final approval for publication.

## DATA ACCESSIBILITY

Data available from the Dryad Digital Repository: https://doi.org/10.5061/dryad.8qm8r4m.
