# Supplementary material for: Variations in age‐ and sex‐specific survival rates help explain population trend in a discrete marine mammal population
Source: Ecol Evol. 2018 Dec 19;9(1):533–44. doi: 10.1002/ece3.4772 (PMC6342117; doi:10.1002/ece3.4772)
Supplement: Supplementary file 1 [file ECE3-9-533-s001.docx]

**Supporting information**

Table S1. Model selection diagnostics for the robust design models to estimate juvenile/adult survival probability (φ), capture probabilities (p), temporary emigration probabilities (ɣ), and Pledger’s mixture parameter for two types (pi). In the model description: (.) = constant; (t) = time-specific for primary occasions (year); (txs) = time-specific for primary (year) and secondary (months) sampling occasions; (trend) = time trend; for temporary emigration ɣ`` = ɣ` = 0 = no emigration; ɣ``(x) = ɣ`(x) = random emigration; ɣ``(x) ɣ`(x) = Markovian emigration. Models are ordered from smallest to largest QAICc.

| Model no. | Model | No. parameters | QAICc | ΔQAICc | QAIC_c_ weight | Q-deviance |
| --- | --- | --- | --- | --- | --- | --- |
| 1 | φ_(trend)_ γ^’’^ _(.)_ γ^’^ _(t)_ p_(txs)_ p_i_ | 191 | 618.87 | 0.00 | 0.989 | 5246 |
| 2 | φ_(.)_ γ^’’^ _(.)_ γ^’^ _(t)_ p_(txs)_ p_i_ | 190 | 628.43 | 9.57 | 0.008 | 5258 |
| 3 | φ_(.)_ γ^’’^= γ^’^= 0 p_(txs)_ p_i_ | 188 | 630.70 | 11.84 | 0.003 | 5265 |
| 4 | φ_(.)_ γ^’’^_(.)_ = γ^’^ _(.)_ p_(txs)_ p_i_ | 189 | 646.08 | 27.21 | 0.000 | 5278 |
| 5 | φ_(.)_ γ^’’^ _(.)_ γ^’^ _(t)_ p_(txs)_ p_i_ | 214 | 652.01 | 33.14 | 0.000 | 5228 |
| 6 | φ_(.)_ γ^’’^_(t)_ = γ^’^ _(t)_ p_(txs)_ p_i_ | 213 | 652.85 | 33.98 | 0.000 | 5231 |
| 7 | φ_(.)_ γ^’’^_(t)_ γ^’^ _(.)_ p_(txs)_ p_i_ | 214 | 658.16 | 39.29 | 0.000 | 5234 |
| 8 | φ_(.)_ γ^’’^_(t)_  γ^’^ _(t)_ p_(txs)_ p_i_ | 238 | 673.86 | 54.99 | 0.000 | 5195 |
| 9 | φ_(.)_ γ^’’^_(t)_ = γ^’^ _(t)_ p_(txs)_ | 186 | 824.39 | 205.52 | 0.000 | 5463 |
| 10 | φ_(.)_ γ^’’^_(t)_  γ^’^ _(t)_ p_(txs)_ | 211 | 837.76 | 218.89 | 0.000 | 5420 |
| 11 | φ_(.)_ γ^’’^_(t)_ γ^’^ _(.)_ p_(txs)_ | 187 | 838.36 | 219.50 | 0.000 | 5475 |
| 12 | φ_(.)_ γ^’’^_(.)_  γ^’^ _(.)_ p_(txs)_ | 163 | 900.63 | 281.76 | 0.000 | 5590 |
| 13 | φ_(.)_ γ^’’^ _(.)_ γ^’^ _(t)_ p_(txs)_ | 187 | 902.46 | 283.59 | 0.000 | 5539 |
| 14 | φ_(.)_ γ^’’^_(.)_ = γ^’^ _(.)_ p_(txs)_ | 162 | 909.40 | 290.53 | 0.000 | 5601 |
| 15 | φ_(.)_ γ^’’^= γ^’^= 0 p_(txs)_ | 159 | 970.41 | 351.54 | 0.000 | 5668 |

Table S2. Model selection diagnostics for the multi-state models used to estimate sex-specific survival for juveniles/adults (φ), recapture probabilities (p) and transition probabilities ψ (sex, time). In the model description: (.) = constant; (t) = time-specific; (trend) = time trend (additive or interaction); (sex) = sex-specific; M = male; F = female; U = Unknown sex. Models are ordered from smallest to largest AICc.

Only the 11 most supported multi-state models are shown (the remaining 33 models received much less support).

| Model no. | Model | No. parameters | AICc | ΔAICc | AIC_c_ weight | Residual deviance |
| --- | --- | --- | --- | --- | --- | --- |
| 1 | φ_(M=U≠F)_ p_(t)_ ψ_(., t)_ | 47 | 4159.80 | 0.00 | 0.297 | 3364 |
| 2 | φ_(M=U≠F + trend)_ p_(t)_ ψ_(., t)_ | 48 | 4161.55 | 1.74 | 0.124 | 3364 |
| 3 | φ_(.)_ p_(t)_ ψ_(sex, t)_ | 46 | 4161.57 | 1.77 | 0.123 | 3368 |
| 4 | φ_(sex)_ p_(t)_ ψ_(., t)_ | 48 | 4161.89 | 2.09 | 0.104 | 3364 |
| 5 | φ_(sex : trend)_ p_(t)_ ψ_(., t)_ | 48 | 4162.07 | 2.27 | 0.096 | 3365 |
| 6 | φ_(trend)_ p_(t)_ ψ_(., t)_ | 47 | 4162.84 | 3.04 | 0.065 | 3367 |
| 7 | φ_(F=U≠M)_ p_(t)_ ψ_(., t)_ | 47 | 4163.63 | 3.83 | 0.044 | 3368 |
| 8 | φ_(sex + trend)_ p_(t)_ ψ_(., t)_ | 49 | 4163.67 | 3.87 | 0.043 | 3364 |
| 9 | φ_(F : trend, M=U(.))_p_(t)_ ψ_(., t)_ | 49 | 4163.93 | 4.13 | 0.038 | 3364 |
| 10 | φ_(F≠U : trend, M(.))_p_(t)_ ψ_(., t)_ | 49 | 4164.20 | 4.40 | 0.033 | 3365 |
| 11 | φ_(sex : trend)_ p_(t)_ ψ_(., t)_ | 49 | 4164.21 | 4.40 | 0.033 | 3365 |

Table S3. Model selection diagnostics for the CJS models used to estimate calf survival and recapture probability. In the model description: (.) = constant; (t) = time-specific; (age) = 1^st^, 2^nd^, 3^rd^ year; (age 1≠2=3) = 1^st^ and 2^nd^/3^rd^ year; (order) = first-born versus subsequently-born calves. Models are ordered from smallest to largest AICc.

| Model no. | Model | No. parameters | AICc | ΔAICc | AIC_c_ weight | Residual deviance |
| --- | --- | --- | --- | --- | --- | --- |
| 1 | φ_(age)_ p_(.)_ | 5 | 236.17 | 0.00 | 0.314 | 137 |
| 2 | φ_(age 1≠2=3)_ p_(.)_ | 4 | 236.66 | 0.49 | 0.246 | 140 |
| 3 | φ_(.)_ p_(.)_ | 2 | 237.30 | 1.13 | 0.179 | 144 |
| 4 | φ_(order, age)_ p_(.)_ | 6 | 237.66 | 1.49 | 0.149 | 136 |
| 5 | φ_(order, age 1≠2=3)_ p_(.)_ | 5 | 238.22 | 2.05 | 0.113 | 139 |
| 6 | φ_(age)_ p_(t)_ | 29 | 270.86 | 34.69 | 0.000 | 116 |
| 7 | φ_(age 1≠2=3)_ p_(t)_ | 28 | 271.19 | 35.02 | 0.000 | 119 |
| 8 | φ_(order, age)_ p_(t)_ | 30 | 272.90 | 36.73 | 0.000 | 116 |
| 9 | φ_(order, age 1≠2=3)_ p_(t)_ | 29 | 273.24 | 37.07 | 0.000 | 119 |
| 10 | φ_(age 1≠2=3)_ p_(age1≠ 2=3, t)_ | 30 | 274.49 | 38.32 | 0.000 | 117 |
| 11 | φ_(order, age 1≠2=3)_ p_(age 1≠2=3, t)_ | 31 | 276.55 | 40.38 | 0.000 | 117 |
| 12 | φ_(age)_ p_(age, t)_ | 32 | 276.60 | 40.43 | 0.000 | 114 |
| 13 | φ_(order, age)_ p_(age, t)_ | 33 | 278.68 | 42.51 | 0.000 | 114 |
